# Supplementary material for: Preparation of CdTe/Alginate Textile Fibres with Controllable Fluorescence Emission through a Wet-Spinning Process and Application in the Trace Detection of Hg2+ Ions
Source: Nanomaterials (Basel). 2019 Apr 8;9(4):570. doi: 10.3390/nano9040570 (PMC6523845; doi:10.3390/nano9040570)
Supplement: Supplementary file 1 [file nanomaterials-09-00570-s001.pdf]

1. Fluorescent properties of three CdTe/SA blends

Properties of MEA-CdTe/SA blends:

In fact, MEA-CdTe NCs and SA formed non-uniform fluorescent gel instead of stable solution (Fig. S1). The gel was induced by the strong electrostatic interactions between the positive $–NH_3^+$ of MEA-CdTe and the negative $–COO^-$ of SA chains.

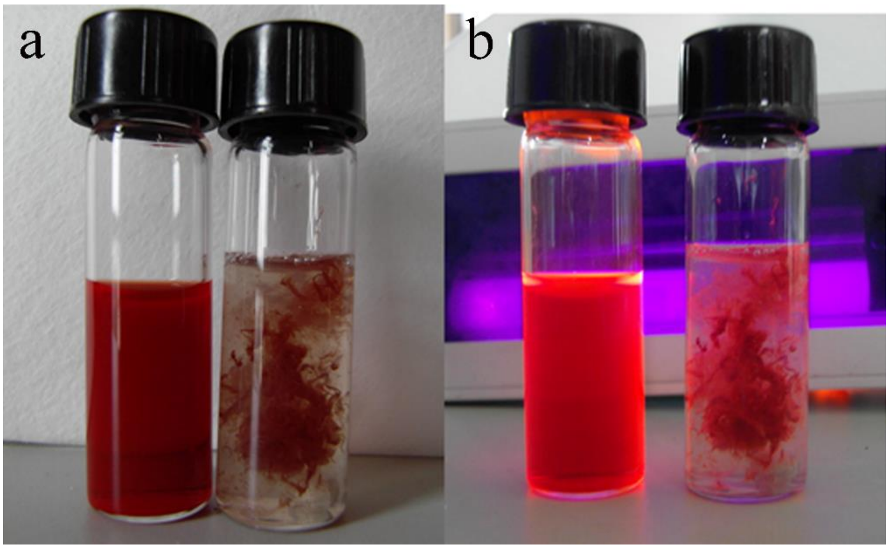

Fig. S1. Optical photos of both the MEA-CdTe NCs dispersion (the left bottle) and the CdTe/SA blend (the right bottle) under (a) daylight and (b) UV-light.

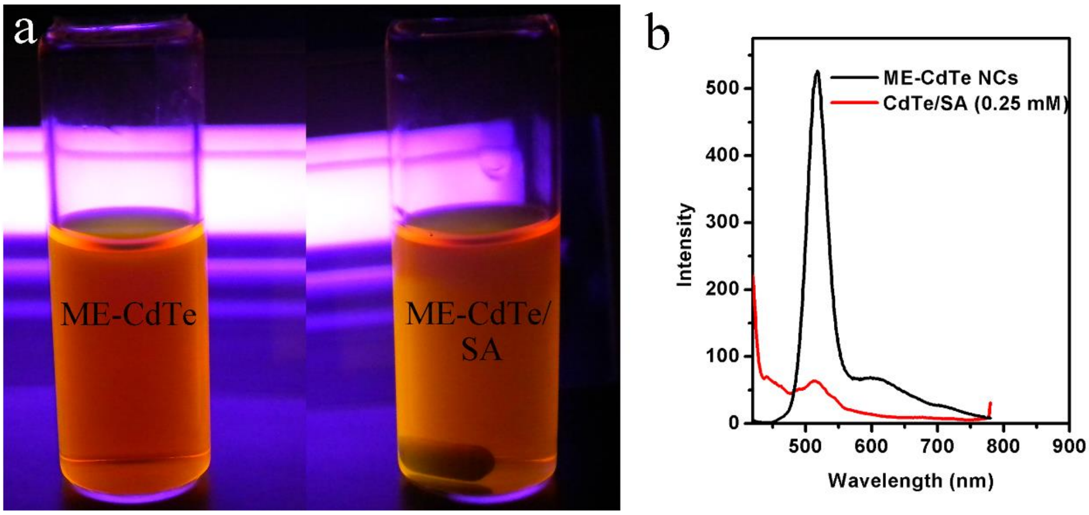

Fig. S2. (a) Optical photos of the ME-CdTe NCs dispersion and the ME-CdTe/SA blend (in which the ME-CdTe concentration is 0.25 mM) under UV-light. (b) PL spectra of the ME-CdTe NCs dispersion and the ME-CdTe/SA blend after stored in the dark for 30 days.

Properties of ME-CdTe/SA blends:

For the ME-CdTe and SA blend, the new prepared blend was a uniform liquid instead of the non-uniform gel formed by MEA-CdTe and SA. As shown in Fig. S2a, under the UV-irradiation, the blend emitted the same bright color with the ME-CdTe NCs dispersion, meaning the ME-CdTe NCs were well dispersed in the blend and their surface ligands were also well preserved. However, as shown by the fluorescent spectra in Fig. S2b, after been stored for 30 days, the emission intensity of the blend decreased dramatically compared to that of ME-CdTe NCs, indicating the CdTe NCs were influenced and even quenched by the SA molecules. As a result, the blending of ME-CdTe and SA were not stable enough to form a stable SD.

Properties of ME-CdTe/SA blends:

TGA-CdTe NCs were finally chosen by testing the stability of TGA-CdTe and SA blend. Fig. S3 showed the PL spectra of the blend which have been stored for 30 days and the original CdTe dispersion. The peak position were the same and the intensity did not change much, which benefited from the static repulsion between the both ionized negatively charged carboxyl groups of TGA-CdTe NCs and SA chains in neutral or slightly alkaline aqueous solution, which also enabled the coexistence and stability of NCs and SA in the solution.

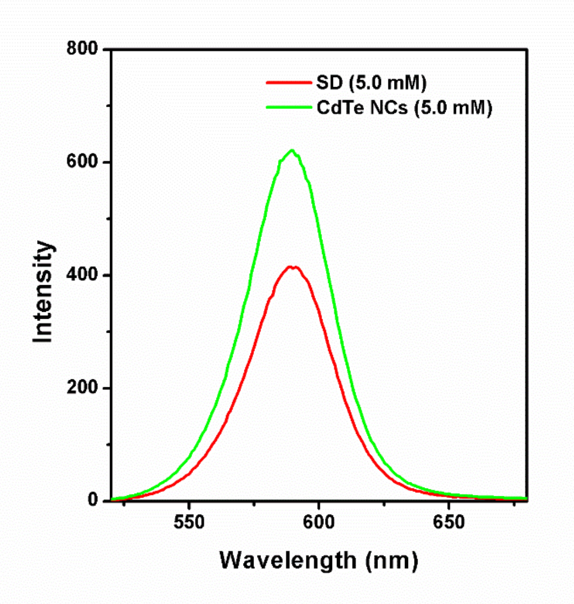

Fig. S3. PL spectra of ThGA-CdTe NCs dispersion and ThGA-CdTe/SA SD with the same NCs' concentration after been stored for 30 days.

2. Rheological properties of TGA-CdTe NCs and SA blends

We further tested the rheological properties of NCs and SA blends with different NCs-concentrations (0, 1.25, 2.5, 5.0 mM, respectively). All the steady state rheological curves in Fig. S4a showed the typical shear-thinning phenomenon of polymer fluid: viscosity (η) was almost constant in the low shearing rate region, and significantly decreased in the high shearing rate region. Compared with the pure SA solution, η of blends slightly reduced due to the increased ionic strengths of solutions induced by the addition of negative charged NCs and their positive counter-ions Na^+. Fig. S4b showed the linear viscoelastic properties of blends and the pure SA solution. All curves followed the same rule: storage modulus (G') < loss modulus (G'') in low frequency region, and $G'>G''$ in high frequency region, which meant the solutions changed gradually from viscous fluid to elastomer with the growing shearing frequency. In addition, G' and G'' of blends were a little lower than the pure SA solution, and the reduced amplitude of G' is more than G''. The intersections of G' and G'' also appeared in

the larger frequency according to the CdTe concentrations. Evidences proved that the addition of CdTe NCs increases the viscous proportion of the SA solution, which increased the spinning ability of SA in some extent.

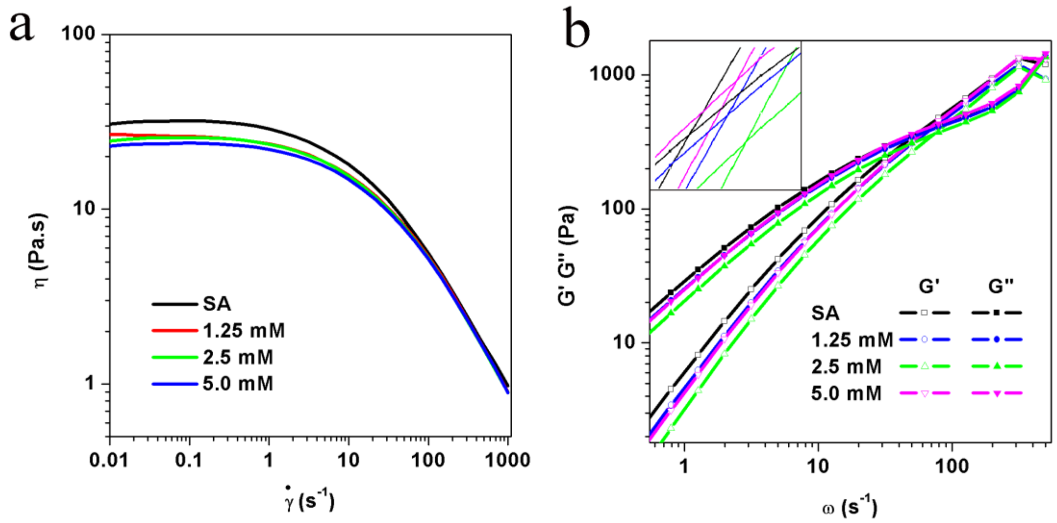

Fig. S4. Rheological properties of SDs and the 4wt% SA solution at 50 °C: (a) shearing rate ($\dot{\gamma}$) dependence of viscosity (η); (b) angular frequency dependence (ω) of storage modulus (G') and loss modulus (G″). Inset: the magnification of the intersection of G' and G″.
